# Spectrum of Tendon Pathologies: Triggers, Trails and End-State

**DOI:** 10.3390/ijms21030844

**Published:** 2020-01-28

**Authors:** Sara Steinmann, Christian G. Pfeifer, Christoph Brochhausen, Denitsa Docheva

**Affiliations:** 1 Experimental Trauma Surgery, Department of Trauma Surgery, University Medical Center Regensburg, Am Biopark 9, 93053 Regensburg, Germany; sara.steinmann@ukr.de (S.S.); christian.pfeifer@ukr.de (C.G.P.); 2 Department of Trauma Surgery, University Medical Center Regensburg, Franz-Josef-Strauss-Allee 11, 93053 Regensburg, Germany; 3 Institute of Pathology, University Regensburg, Franz-Josef-Strauss-Allee 11, 93053 Regensburg, Germany; christoph.brochhausen@ukr.de; 4 Department of Medical Biology, Medical University-Plovdiv, 15A Vassil Aprilov Blvd., 4002 Plovdiv, Bulgaria

**Keywords:** tendon pathologies, tendinopathy, tendinitis, tendinosis, tendon rupture, risk factors, tendinopathy management

## Abstract

The biggest compartment of the musculoskeletal system is the tendons and ligaments. In particular, tendons are dense tissues connecting muscle to bone that are critical for the integrity, function and locomotion of this system. Due to the increasing age of our society and the overall rise in engagement in extreme and overuse sports, there is a growing prevalence of tendinopathies. Despite the recent advances in tendon research and due to difficult early diagnosis, a multitude of risk factors and vague understanding of the underlying biological mechanisms involved in the progression of tendon injuries, the toolbox of treatment strategies remains limited and non-satisfactory. This review is designed to summarize the current knowledge of triggers, trails and end state of tendinopathies.

## 1. Terminology of Tendon Diseases

Tendon disorders have become very common in today’s athletic and non-athletic population and account for a substantial proportion of activity-related diseases of the musculoskeletal system. Due to the increasingly aged demographics of our society with elevated life expectancy and a rise in engagement of young people in extreme and/or competitive sports, tendinopathies and subsequent tendon ruptures present a major clinical and financial challenge in modern medicine.

Tendinopathy, the pathological change in the tendon that can be classified as a failure in homeostatic response of the tendon, is a debilitating condition mainly occurring in the active workplace and in the sports field, which can end in integrant morbidity and disability. The blanket term “tendinopathy” is used to describe a broad spectrum of non-rupture clinical burdens affecting tendons of primary, acute and chronically degenerative tendon pathologies, which are associated with prolonged pain, impaired performance, swelling and further pathological characteristics [1]. Currently, tendinopathies are divided into three groups, “tendinosis”, “tendinitis” and “tenosynovitis” [2]. “Tendinosis” describes preferentially chronic degenerative conditions of the mid-substance tendon resulting from an accumulation of micro-trauma over time, devoid of inflammatory impact and form the most pathological disorders affecting tendons [1,3]. The term “tendinitis” has been originally employed to designate any painful tendon impairment, acute or chronic, associated with intra-tendinous inflammation and presence of inflammatory cells [1,3,4]. “Tenosynovitis” (also “paratendinitis” and “peritendinitis”) describes the involvement of the paratenon (paratendinopathy) alone or in combination with tendinosis. This term refers to inflammation of the tendinous sheath and is strictly speaking not a classical tendinopathy in which degeneration is observed within the tendon itself. Finally, spontaneous tendon tears and ruptures without prior symptoms are summarized as tendon injuries [2] and form the final condition of tendinopathies that failed to heal, thus resulting in loss of the tendon continuum.

So far, there is no reliable method to detect early tendinopathy and no strategy to ameliorate its progress. Inevitably, tendinopathies lead to tendon rupture and once this happens, tendon natural healing is slow, often poorly responding to treatments and requires prolonged rehabilitation in most cases. Until today, none of the therapeutic options offers satisfactory long-term solutions, meaning that repaired tendons do not regain their complete strength and functionality [5]. However, the understanding of tendon biology, degenerative and healing processes, progresses slowly and the development of new treatment options is therefore insufficient. In this review, we aimed to summarize the current state of knowledge on tendinopathies including potential triggers, trails, end-state and treatment options.

## 2. Tendon Structural and Functional Relationship

Tendons and ligaments play a crucial role in the locomotive system [6,7,8,9]. In the human body, there are nearly 4000 different tendons and ligaments. Specifically, tendons are dense connective tissues and critical components for body posture, integrity and function of the musculoskeletal system, as they connect muscle (musculotendinous junction) to bone (enthesis) to buffer and transmit forces on which locomotion is entirely dependent [6,7]. In addition, tendon- or ligament-like structures exist in some peculiar places, which we often forget about; in the heart (chordae tendineae cordis connecting the valves to the heart muscle), in the diaphragm (central tendinous region), in the ear’s hearing apparatus (e.g., stapedial tendon) and in the vocal apparatus (vocal ligaments within the vocal folds).

**Figure 1:**
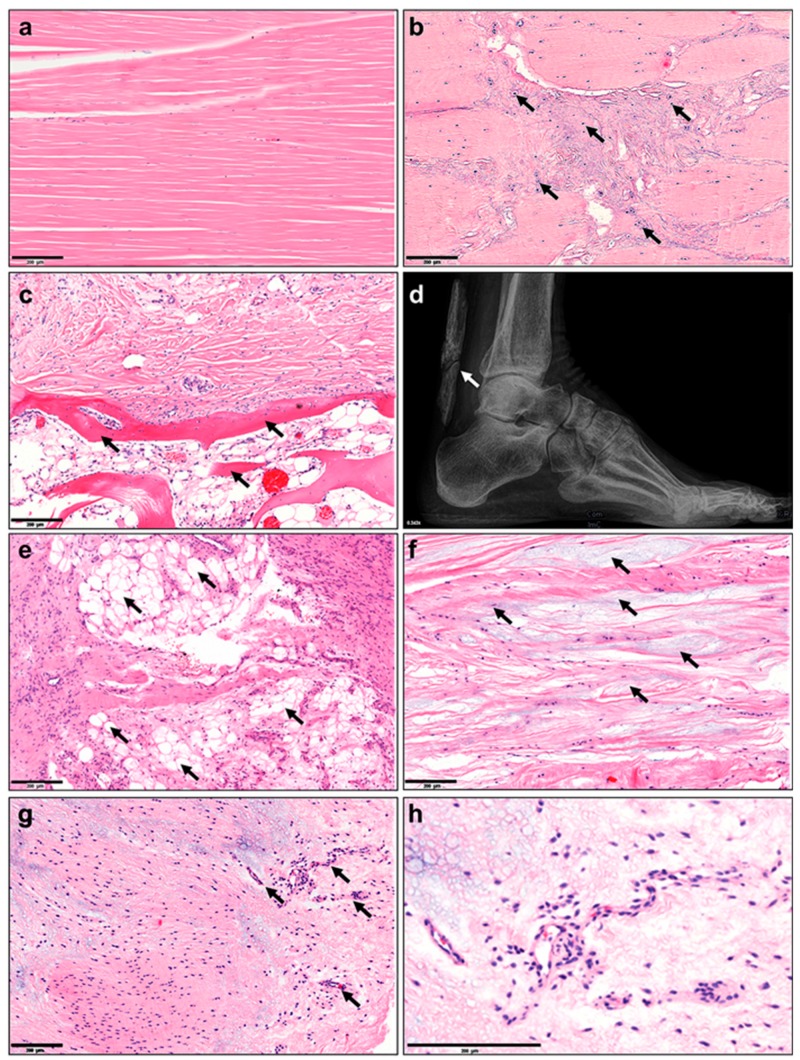
Histopathological changes in tendinopathy. (**a**) Normal tendon tissue, (**b**) fiber crimping and kinking, loosening of collagenous matrix, (**c**) increased proteoglycan (PG)/glycosaminoglycan (GAG) production and changed cytokine profiles, (**d**) hypercellularity, (**e**) apoptosis, (**f**) presence of other cell types such as chondrocytes (fibrochondrogenesis), (**g**) osteocytes (calcification), h) adipocyte accumulation, i) hypervascularization and j) innervation. Based on Järvinen et al. [25].

**Figure 2:**
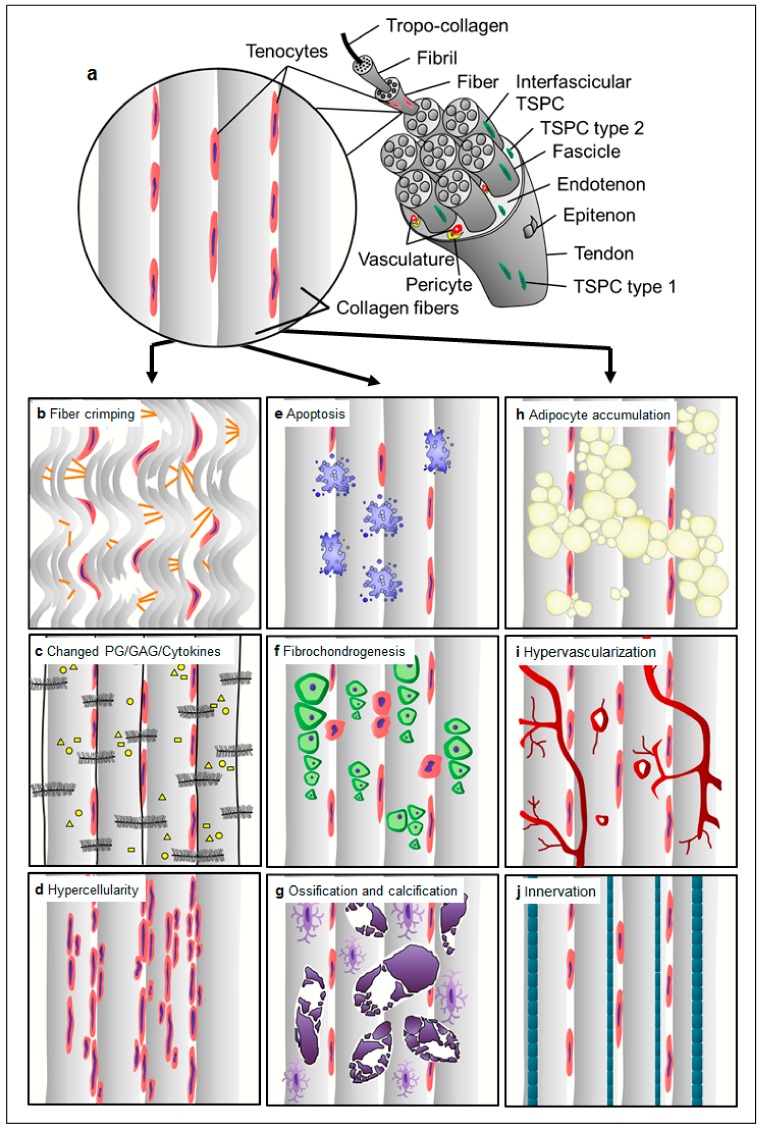
Tendinopathy-related histopathological characteristics in human tendon tissue. Hematoxylin–eosin staining of (**a**) normal tendon tissue and abnormalities such as the (**b**) presence of chondrocytes, (**c**) ossification, (d) calcification of the Achilles tendon (X-ray image), (**e**) accumulation of adipocytes, (**f**) myxoid (or mucoid) degeneration and (**g**) hypervascularization. (**h**) Image g) 10 x zoomed in. Scale bars: 200 µm. Arrows indicate the corresponding feature per image. Images derived from the histopathological archive of Prof. Christoph Brochhausen, Institute of Pathology, University Regensburg.

Tendons are highly structured dense connective tissues which provide stabilization of joints and are able to store and release elastic energy that allows movement [8,9]. They are mainly composed of collagen fibers and tendon-resident cells which lie embedded in parallel rows (Figure 1a, Figure 2a) in a well-organized extracellular matrix (ECM), containing high amounts of proteoglycans. The collagen fibers confer the tissue biomechanical strength and resistance to tension, whilst the proteoglycans provide the viscoelastic properties for the tendon [8,9]. Cross-linked tropocollagen forms insoluble collagen molecules that aggregate into increasing order of microfibrils, fibers, bundles and fascicles [8,9]. The fascicles are ensheathed by a thin layer of loose connective tissue, known as the endotenon, enabling them to glide and extend to each other. Several fascicles form the whole tendon unit and are covered by another sheet—the epitenon, a dense fibrillary network of collagen preventing adhesion to the neighboring tissues [8,9]. This internally hierarchical structure enables the tissue to have a high tensile force and resilience but also prevents quick damage, whilst the endotenon and epitenon sheets on one side prevent facile separation under mechanical stress and on the other side, they carry blood vessels, nerves and lymphatics to deeper portions of the tendon unit [8,9]. The paratenon is the very outer layer and functions to reduce friction between the tendon and nearby tissues [8,9].

The tendon-resident cell population is composed of approximately 90%–95% of tenocytes (Figure 1a) which are terminally differentiated and no self-renewable cells of elongated shape that produce the tendon ECM [6,7]. The remaining 5%–10% of cells in the tendon are tendon stem/progenitor cells (TSPCs) and tenoblasts, which can proliferate and have the potency to differentiate towards other mesenchymal lineages and are involved in the tissue repair processes [10]. Their subtypes, the stage of differentiation and the precise location are still not fully defined due to the lack of gene markers, that allow precise segregation. In addition, chondrocytes are found in compressive areas including the enthesis at the junction to bone. Furthermore, endothelial-, perivascular-, smooth muscle- and neuronal cells are found in the endotenon and epitenon sheets, where vasculature and nerve fibers run [6,7].

Loading and compressive forces regulate the production and degradation of ECM whereas proteoglycans enable homeostasis of the tendon tissue. In conclusion, it is important to understand the complex interplay of loading, structural changes and cell response in healthy tissue when considering treatment of injured tendons.

## 3. Tendinopathy: A Challenging Disease

At present, the underlying pathology of tendinopathies is not well defined and their etiology and epidemiology are basically neither rationally determined nor generally valid. The difficulty lies within their highly heterogeneous nature which is suggested to be coherent with the multitude of potential risk factors that are considered to trigger tendon disorders and the wide range of degree. Furthermore, individual patient characteristics (e.g., age, gender and genes), habits (e.g., alcohol consumption and smoking) and activity levels contribute to the diverse spectrum of tendinopathies. Moreover, the variety of symptoms such as pain, tenderness and rigidity, swelling, bulge formation within the tendon, redness, warmth, muscle weakness and myalgia, spasticity, cramping and even asymptomatic conditions provide challenges, since they do not appear uniformly but vary from case to case. Curative and tailored management relies not only on full understanding of the tendon pathogenesis but also on appropriate, widely accepted and guiding classification and staging systems, which would be beneficial for rational and adequate patient-targeted therapy. Based on clinical and basic scientific studies in humans, Cook and Purdam (2009) established the continuum theory for pathogenesis of tendinopathy from asymptomatic tendons to tendon injuries claiming that tendon pathogenesis is a continuum, not an absolute [11]. This concept provides the heterogeneous staging of tendon pathology and suggests that in response to an acute injury or micro trauma, tendons in the progress of pathogenesis undergo a cascade of three phases from normal tendons to tendon tear and rupture. These phases are 1) early reactive tendinopathy (non-inflammatory proliferative response in cells and matrix) in response to acute overload or trauma, 2) failed healing response and disrepair of the ECM and finally, 3) terminal degeneration and dysregulation of healing resulting in irreversible stage of pathology showing major structural and compositional changes, cell death, tissue breakdown and loss of function with predisposition of the tendon to further injury and rupture [11]. One accepted key hypothesis is that the interactions between tendon cells and their mechanical environment might be deterministic for the pathogenesis of tendons. Here, the authors tried to incorporate existing clinical, histological and imaging information of tendon pathology with the aim to develop a model that can be continually evaluated and modified according to novel research findings. To date, this model offers high potential to provide the basis for individual assessment and targeted treatment aligned to the stage of pathology. Due to failing of proper staging using current error-prone imaging technologies, classifying cases to specific tendon disease stage is imperfect and the detailed tendon pathology and mechanisms per stage still remain unknown.

## 4. Morbidity and Clinical Relevance of Tendinopathies

Tendon disorders are medical conditions including traumatic or overuse injuries and a spectrum of inflammatory and degenerative tendon changes [7]. Patients affected by acute or chronic tendon disorders suffer from pain, immobility and as a consequence, a loss of daily activities as well as sport activities. While tendon ruptures present with sudden onset of pain and loss of function, chronic tendon diseases often appear with slowly elevating pain and steadily increasing loss of function. Due to this creeping onset, patients are often diagnosed delayed, treated inadequately and thus, suffer in the longer term [7].

In general, tendon disorders may be attributed to abnormal tendon loading, abnormal ECM composition and malfunction alongside altered biological and genetic variations that may consequently lead to exceedance of tendon’s capacity followed by ruptures [7]. Extrinsic factors such as certain medications, irradiation or infiltration can cause deterioration of tendons’ biomechanical capabilities [7].

Tendon injuries do not only occur in physically active adults and adolescents but they also appear among the population with a moderate physical activity. Depending on the injury site, tendon injuries are even more frequent in elderly and more inactive patients, as seen in rotator cuff tears [12]. Sex specific injury patterns and incidence can also be observed according to the affected tendon. Noncontact anterior cruciate ligament (ACL, a tendon-like structure) injury, for instance, was associated with gender-disparity showing alarmingly higher rate in female athletes [13]. In contrast, Achilles tendon ruptures are more common in men [14]. Tendinopathies are typically classified by their location since dependent on the site of injury they exhibit different pathomorphologies and require different treatment approaches. These are tendinopathies of the upper extremities (e.g., rotator cuff tendinopathy and biceps tendinopathy) and lower extremities (e.g., patellar tendinopathy and Achilles tendinopathy) [15].

Tendon disorders can occur in any region of the tendon: the tendon insertion, mid tendon substance, musculotendinous junction or tendon sheath. For example, Achilles tendon ruptures are diagnosed in 84,000 Europeans annually, with 30% of these being surgically treated [16], and they usually occur at the insertion site of the tendon. Another common tendon injury is the painful rotator cuff tear, mainly in the tendon mid-substance that requires surgical repair, affecting approximately 85 people per 100,000 in the general population [17] with dramatically elevated incidence with increasing age. Especially in rotator cuff disease, differentiation between full thickness tears, partial articular sided tears, e.g., Partial Articular Supraspinatus Tendon Avulsion (PASTA) lesions, tendon retraction and muscle quality is mandatory for correct choice of treatment. However, treatment options in other pathomorphologies differ depending upon various factors that can be distinguished into patient immanent factors, injury characteristics and patient extrinsic factors. In the recent decades, the prevalence of, for example, Achilles tendinopathies and ruptures has risen due to both an increase in the elderly population and a higher participation in excessive physical activities [18]. In the general population, the incidence of Achilles tendon ruptures, a typical injury among 30–50 year-old men, is up to 1%. Achilles tendinopathies appear more frequently, with a lifetime risk of 52% in former elite male runners and the incidence accounts for 5.9% among sedentary people, 24% among competitive athletes and 18% among athletes younger than 45 years. Generally, Achilles tendon ruptures occur with different frequencies at various anatomical locations: 75% at the mid-substance, 10%–20% at the distal enthesis, and 5%–15% at the myotendinous junction [19]. According to current trends, Patellar tendinopathy is another common load-induced injury with a prevalence of 45% in volleyball players and 32% in basketball players [20]. Lateral epicondylitis, which is also known as tennis elbow, is a frequent injury that not only occurs in athletes but also in the general population with an incidence of 4–7 people per 1000 per year [21]. Rotator cuff injury runs the full spectrum from injury to tendinopathy to partial tears, and finally complete tears. Age plays a significant role. Injuries range from 9.7% in those 20 years and younger, increasing to 50% likelihood of bilateral tears of 60 years old and 62% in patients of 80 years and older (whether or not symptoms were present) [22].

In sum, tendon demographics are very relevant for modern medicine also considering the overall aging of human society and thus adding additional pressure to tendon-related science to provide strategies to improve our current management of tendinopathies.

## 5. Tissue Changes: Histopathological, Structural, Cellular, Epigenetic, Transcriptomic, Proteomic and Metabolomic

At the onset, tendinopathy is characterized by tendon stiffness, impaired function, local swelling and pain. Tendinopathies are exposed by macroscopic failure in the entire tendon structure and material. In contrast to glistening-white healthy tendons, symptomatic tendons appear more amorphous, grey-brown, friable and edematous [23,24].

Tendons are known to adapt their structure depending upon their physiological mechanical environment. Hence, they also respond to pathological stimuli (e.g., risk factors given below) which interfere and deteriorate the primordial tissue homeostasis creating histopathological changes of the entire tissue, a condition, which has been extensively described before in many previous studies and reviews [3,25,26]. To this day, the underlying mechanisms for aberrant rearrangement remain unclear.

In comparison to normal tendon with well-aligned parallel and compact collagen fibers with adjacent tenocytes (Figure 1a, Figure 2a), the most prominent changes occur in the disorganization of the tendon matrix represented by discontinuous, crimped and thinned collagen fibers with loss of their typical hierarchical structure (Figure 1b). Pathological tendons reveal loss of matrix integrity by reduction of total collagen content and increased production of ECM components (proteoglycans and glycosaminoglycans; Figure 1c) that results in tendon stiffening.

Moreover, tendinopathy affects the cell density (hypercellularity; Figure 1d) often being accompanied by areas of enhanced necrotic or apoptotic cell death (Figure 1e) and abnormal possibly senescent tenocyte- and nuclei rounding (Figure 1f). Furthermore, resident TSPCs can respond abnormally to signals changed cytokine and growth factor profiles (e.g., IL-1β, TNFα, MMP-2, -3, -9, -13, COX2, VEGF and TGF-β), and choose entry into alternative non-tenocyte cell fate. Thus, resulting ingrowth of exogenous cell types (Figure 1f–h, Figure 2b–h) and metaplasia formation including cartilaginous, fibrochondroid (Figure 1f and Figure 2b), bony (ossification, Figure 1g and Figure 2c,d), or adipocyte (Figure 1h, Figure 2e) transformation. Occasionally, tendon degeneration also involves mucoid or myxoid degeneration with the development of mucoid fibrin deposit (Figure 2f), as well as hypervascularity (Figure 1i, Figure 2g,h) and innervation [27,28], which may be accompanied by inflammatory response and pain.

In addition to our understating of histopathological, structural and cellular changes, in the last decade, the tendon field has also entered into new research areas such as epigenetics, RNA modifications (transcriptome and regulatory RNAs) and even proteomics and metabolomics.

Recently, intriguing epigenetic alterations were suggested to be associated with tendinopathies. Analysis of promotor methylation of the MMP11 gene has revealed significant difference in the methylation status of a single CpG site (highly frequent regions of DNA, where a cytosine nucleotide is followed by guanine) 65 base pairs upstream of the MMP11 promoter between patients with patellar tendinopathy and healthy controls. In turn of the hypermethylation of the MMP11 promotor, the authors speculated that MMP11 substrates would accumulate in the tendon matrix as the amount of the MMP11 enzyme available for catalysis would be limited. The misbalance of the protein composition of the tendon matrix will thereby lead to degenerative progression [29,30]. Altered methylation status of CpG islands upstream of the ADAMTS4 gene, but not the TIMP2 gene, was also associated with human patellar tendinopathy [29,30]. Review analysis of Vitamin C, a known critical cofactor of collagen synthesis, has also proposed that this vitamin can regulate epigenetic signatures by enhancing the activity of DNA and histone demethylases in the cell nucleus, thus steering a cell reprogramming towards more pluripotent state, which may influence the ECM/collagen homeostasis also in the tendon. However, it remains to be validated experimentally, whether Vitamin C-dependent epigenetic changes influence tendon physiology and disease.

Transcriptome comparison of aged/degenerative TSPCs to young/healthy TSPCs has shown a profound shift in their gene expression. Gene ontology analysis of top significantly dysregulated genes have suggested two main clusters of gene function to be affected: namely, resistance to and clearance of cellular stress, cell–cell and cell–matrix communication and cytoskeletal dynamics [31]. A follow up study of the microarray data by Popov et al. has shown that several members of the ephrin receptor family, important for cell–cell signaling, are significantly downregulated in aged/degenerative cells but interestingly, reconstituted some cellular features such as self-renewability and wound healing potential are greatly improved. Cross platform analysis of transcriptomic data investigated connections between age, gender and sex hormones during the development of tendinopathy and identified that in old males decreased expression of CRABP2 leads to cell proliferation, whereas in old females it leads to cellular senescence [32]. This study gives the important notion that tendon degenerative diseases may need to be treated differently in males and females because alternative mechanisms may be involved [32]. Moreover, a number of studies based on RNA sequencing were recently released, revealing higher cellular heterogeneity in tendon tissues than ever expected. Using healthy and diseased tendon samples and then carrying out single cell gene expression analysis, five distinct tenocyte populations, in addition to endothelial, T- and macrophage cells, were discovered [33]. One group was enriched with microfibril-associated genes; second was Scleraxis positive, co-expressing high levels of pro-inflammatory factors; third was a population of fibro-adipogenic progenitors; fourth, TPPP3/PRG4 double positive chondrogenic group and lastly, smooth muscle mesenchymal cells [33]. The authors also reported that in tendon pathology, there are changes in these cell subpopulations urging future studies on the precise cellular interplay [33]. Again, based on single cell transcriptomics and lineage tracing another TPPP3 positive cell fraction co-expressing PDGF receptor alpha was found and suggested to be acting as resident stem cells in the tendon, which can generate tenocytes during healing [34]. Interestingly, fibro-adipogenic progenitors were also identified in this study and suggested to give rise to fibrotic cells and scars during tendon repair. It will be of interest to compare the TPPP3/PDGF receptor alpha double positive cell to Nestin high population found by Yin et al., who proposed that these cells are also stem-like with strong tenogenic potential [34,35]. Transcriptomic studies of mouse Achilles identified 13 unique cell types including four previously undescribed populations of fibroblasts. Another group proposed that there is also a regional impact, tendon proper and peritenon, on the tendon subpopulations and their gene profiles. This uncovered tendon cell heterogeneity may have important implications for our understanding of how tendon tissue is assembled and maintained in physiology and disease, as well as contributing to the design of therapies to treat tendinopathies.

In addition to transcriptome analyses, further understanding of post-transcriptional regulation of gene expression in tendon physiology, disease and repair is also of great importance. Here, identifying non-coding RNAs such as micro RNA, long- and short-non coding RNAs and examining their expression and function in tendinopathy will be of relevance [36]. Numbers of studies have initiated investigation of specific microRNAs in relation to tendon degenerative diseases and reported promising novel traits [36,37,38,39,40,41,42].

Wide characterization of the protein composition and alteration in tendon disease can be gained via proteomics; however so far, only a few studies focusing on tendon tissue were reported. One group performed profiling of young, aged and injured tendons and found that young tendons contain more protein fragments than the aged, suggestive that the protein turnover decreases with age [43]. Furthermore, the authors found distinct regional protein composition of the fascicular and interfascicular matrix and proposed that the interfascicular matrix has higher turnover [44]. Furthermore, proteomics of human supraspinatus disease identified marked protein changes to the elastic fiber, fibrillin-rich and pericellular matrix niches [45]. Additional information related to protein synthesis and degradation can be yield by metabolomics. Metabolomic profiling of patients with rotator cuff stiffness and tears showed interesting changes in lipid-related metabolites indicating cholesterol levels may be related to the rotator cuff pathogenesis [46]. Glucose and lactate metabolisms have been discovered to play a role in tendon repair as well as in tenogenic differentiation via metabolomic analysis, making this area of research also attractive for further expansion into tendon-related studies [47,48].

A study released in 2020, dealing with mechanical loading response in hypervascular tendon, demonstrated a rapid cell-mediated tissue breakdown upon mechanical unloading, in contrast to unloaded physiologically normal hypovascular tendon [49]. Analyses of tissue transcriptome and secretome revealed that a stromal niche with elevated tissue oxygenation and temperature drives a ROS-mediated cellular stress response that leads to adoption of an immune-modulatory phenotype within the degrading stromal tissue [49]. Degradomic analysis further showed a surprisingly rich set of active matrix proteases behind the progressive loss of tissue mechanics [49]. This study is an example of how integrating different large data sets may help us to draw more accurately the interdependent and complex scenario behind tendon pathophysiology.

Altogether, these new investigatory lines should be followed, as they may yield not only breakthrough molecular discoveries underlying certain tendinopathies, but also may help us gain a more integral picture of the different levels: tissue, cellular, DNA, RNA and protein levels, as well as different regulatory programs affected during progression of tendinopathy.

## 6. Vasculature, Inflammation and Neurons

The role of vasculature and inflammation in tendinopathy has been controversial and patchy in literature. Neovessels in the pathogenesis of tendinopathy are still poorly understood [50]. These vessels differ from vasculature formed during development and tissue growth; they leak and do not have proper perfusion [50]. Thus, neovessels fail to deliver oxygen and nutrients to tissue regions under hypoxia [50]. Järvinen et al. [50] has recently postulated a new model for vascular pathogenesis in tendinopathy where the cells in tendinopathic tendons respond to prevailing hypoxia by secreting growth factors and attracting angiogenesis and inflammatory cell accumulation. One possibility to overcome this problem would be to find strategies to stabilize neovessels to functional blood vessels and thereby returning to normal oxygenation. [50]

Inflammation in the context of tendon repair has been better addressed and it is accepted to be necessary for debridement after injury and activation of endogenous cell response, however persistent inflammation can drive fibrosis and thereby compromise the healing outcome [4]. The ability to resolve inflammation by the resident cell populations in tendons at an appropriate time would be crucial for successful outcome. Studies on anti-inflammatory drugs (reviewed in Dakin et al. [4,51]) have reported to induce side effects on the resident cells, as well as on resolving components of the inflammatory response. Therefore, a prolonged use of such drugs may be a contraindication as a therapeutic approach [4]. In this area, it would be of great importance to understand the interplay of immune (macrophages and T-cells) and tendon resident cell populations during the time course of tendon repair [4]. Identifying strategies to influence the presence, activity and depletion of target cell populations may establish a novel approach to steer the repair process to a satisfying outcome [4].

On the contrary to inflammation in tendon repair, a debate remains regarding the role of an inflammatory process in tendinopathy owing to a lack of clinical correlation [52]. Nevertheless, many studies have highlighted the presence of immune cells and inflammatory mechanisms throughout the spectrum of tendinopathy in both animal and human models of disease (reviewed by Millar et al. [52]). In particular, macrophages are becoming more recognized to play a role in tendinopathy. Recently, macrophage activation pathways have been identified in diseased human rotator cuff. At the early stage of the disease, interferons and NF-kB were dominant, whilst at the advanced stage of the disease, STAT6 and glucocorticoid receptor signaling pathways were activated [4,53]. Key inflammatory regulatory molecules, namely cytokines (IL-33), nitric oxide, prostaglandins and lipoxins play a crucial role in modulating changes in the extracellular matrix within tendinopathy [52].

Neuronal changes, in particular glutaminergic alterations, have been associated in painful tendinopathy, while sensory neuropeptides have been linked to failed healing and pain (reviewed in Dean et al. [53]). In clinical terms, glutamate appears important also for tendon healing whilst its upregulation in tendinopathy appears consistent with persistently failed repair response [53]. However, little work had been carried out to carefully investigate the effects of glutamate and other neuropeptides on tendon derived cells and altogether, this area of research is still very fragmented and will require further investigation on in vitro, in vivo and clinical levels.

Further understanding on the links between vasculature, inflammatory mechanisms, neuronal alterations in tendon diseases, as well as during healing, needs to be gathered in order to outline more precisely their impact and to develop novel therapeutics working along these axes for human tendinopathy [52,53].

## 7. Biochemical and Biomechanical Alterations

In the recent decades, biochemical and molecular studies of tendinopathy have advanced our understanding of the underlying degenerative process. It can be considered a failure of matrix adaptation and remodeling due to an imbalance between matrix decomposition and synthesis due to a variety of stresses and mechanical loads. The major structural and molecular changes include: up-regulation of collagen type I and collagen type III mRNA and a shift to a higher collagen type III abundance in relation to collagen type I in the ECM [54], elevated levels of fibronectin, tenascin C, glycosaminoglycans (GAGs), aggrecan and biglycan [55,56]. There are also changes in the activity of various MMPs [29,57,58,59]. The imbalance between matrix metalloproteinases and their endogenous inhibitors (TIMPs) is considered to play a crucial role in the degenerative process [60,61]. Downregulation of MMP-3 and upregulation of MMP-2 and vascular endothelial growth factor (VEGF) was reported in Achilles tendinopathy [62,63,64,65]. Tendinopathy also involves an increase in inflammatory mediators such as prostaglandin E2 and interleukin -1, an enhanced expression of cyclooxygenase 2, growth factors including TGF-β and platelet derived growth factor (PDGF), insulin-like growth factor-1 (IGF-1) and neurotransmitters such as glutamate and substance P [66,67,68,69,70,71,72].

The human tendon has the ability to adapt to loading through an increased collagen synthesis and MMP activity [57]. This adaptation modifies the mechanical strength and the viscoelastic properties and furthermore, decreases the stress susceptibility, which in turn, leads to a higher load resistance. Nevertheless, the tendon has to withstand tremendous forces during repetitive activities making the tendon prone to overuse injuries. Over time, repetitive tensile loading under the tendon injury threshold can lead to an accumulation of micro-injuries, that elevate the risk for tendinopathy and end up in rupture. As a result of micro-injuries, scattered vascular in-growth including necrotic capillaries contributes to vascular compromise [73,74]. This leads to local tissue hypoxia which is considered to increase the risk for degeneration and tendinopathy [75]. Subsequently, aging does not only alter the tendon mechanical properties but also tendon tissue metabolism [46,47,48] and the “fitness” of endogenous tendon cells [31,76,77], a term which is indicative for their native capacity and efficiency of tendon cells to fulfill their basic functions in the tendon such as evading senescence, responding to growth factors and cytokines, maintaining tendon cell fate, matrix homeostasis and proper mechanotransduction. All in all, during aging there is increasing susceptibility for micro-injuries and thereby prevalence of degenerative changes.

## 8. Tendinopathy: Variety of Etiological Factors and Disease Triggers, Trails and End-State

**Figure 3:**
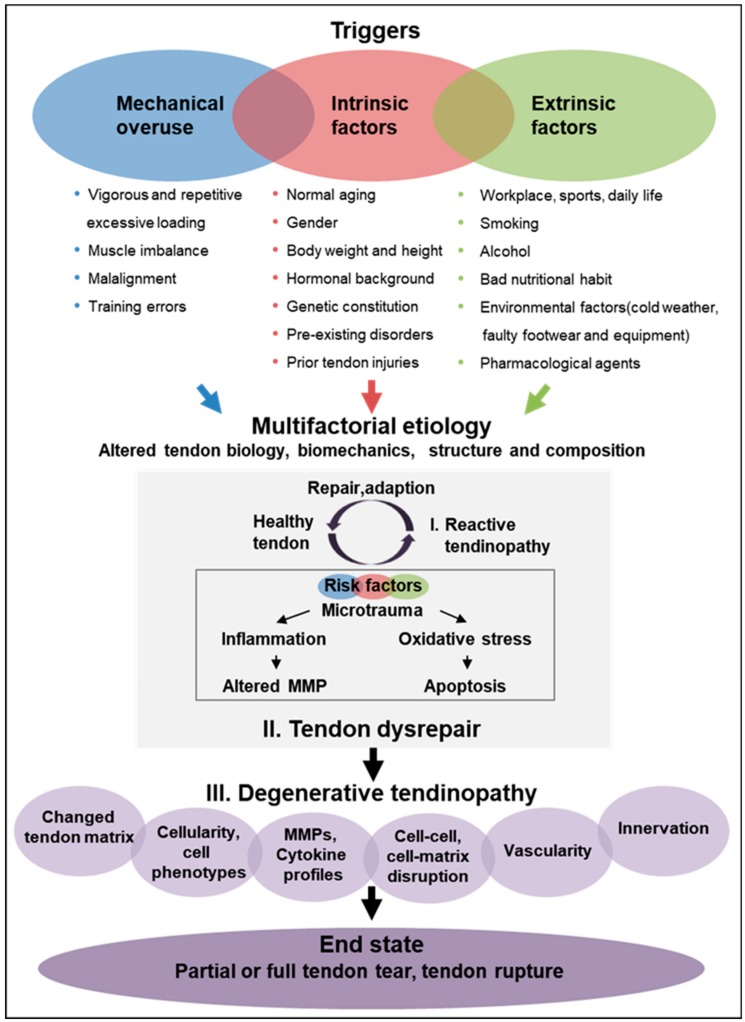
Schematic representation of tendinopathy pathogenesis. It is hypothesized that various risk factors including mechanical overuse as well as intrinsic and extrinsic factors can trigger the development of tendinopathy in a continuous way. The first step is that these risk factors impair proper tendon repair leading to early reactive tendinopathy, which still harbors the capacity for healing. Furthermore, accumulation and increase of risk factors lead to tendon disrepair, that subsequently results in tendon degeneratione. Poor function and load capacity of the tendon finally end up in the end-state of tendinopathy, that leads to tendon tearing or rupture. Based on Cook and Purdam [11] and Shearn et al. [89].

The etiology of tendinopathies is known to be multifaceted (Figure 3) which might be one possibility to explain the different shades of tendinopathy. Traditionally, tendinopathies are proposed to result from acute traumatic load, repetitive mechanical traumas or overuse beyond the capacity of the affected tendon. However, tendinopathies are no longer suggested to be an overuse injury per se, but other promoting factors, alone or in combination, have to be considered as well [78,79,80]. One possibility to explain the different shades of tendinopathy is that diverse triggers can initiate the process, which gradually funnels down to distorted cell–cell and cell–matrix communication and thereby, the inherent cell and matrix organization of the tendon tissue is lost inevitably leading to tendon rupture. In this process, exogenous lineage cells can be activated causing vascular and neuronal in-growth, inflammation and pain but also endogenous tendon stem/progenitor cells can respond abnormally to signals and choose entry into alternative cell fate, thus resulting in tendinous fattening and calcification (Figure 3). The changes occurring during tendinopathy are considered a functional adaptation to the altered mechanical loading. There are three main hypotheses about the reasons for tendon degeneration: (1) mechanical overuse (via matrix), (2) neo-vascularization (via exogenous cells) and (3) cell and tissue aging (via endogenous cells). Most likely, all these three triggers cross-talk and cross–react; however, so far detailed studies on the above have not been carried out in a systematic fashion. Figure 3 illustrates that tendinopathy is a step-wise development starting from initiating risk factors and resulting in the end state of tendon rupture. However, to date the precise speed of disease progression is unknown, since it is impossible so far to diagnose the initial steps due to the lack of technologies to detect the early changes. Moreover, since it is a multifaceted disease, the progression speed might be different, for example, in fattening tendinopathy versus calcifying tendinopathy (Figure 2). The process may also be influenced by age, gender, mobility and overall health status of the patients. In all, “one size fits all” or averaged kinetics of the entire tendinopathy process for human populations might be difficult to obtain.

A variety of biological parameters and lifestyle-related factors as well as pharmacological agents [81] are contemplated to play a major role in the development of chronic tendon pathologies. Here, we distinguished between mechanical overuse- and load-related triggers, intrinsic (acting from within the body) and extrinsic (acting on the body) etiological factors (Figure 3).

Moreover, individual biomechanics such as movement kinetics and kinematics, flexibility, foot posture, neuromuscular capacity and structural anatomy may affect tendinopathy. Tendons become progressively damaged through repetitive excessive loading, change of load or compression. Furthermore, the malalignment and imbalance of surrounding muscle tissue, as well as training errors promote progression risk [53,82,83].

Besides mechanical risks, which are seen as key initial triggers for tendinopathy, multiple systemic (intrinsic) factors, which are very individual, seem to have a relevant impact on tendinopathy. Thus, age and aging, gender, body weight and height, genetics, hormonal background (e.g., menopause), pre-existing disorders (e.g., obesity, hypercholesterolemia, diabetes mellitus, adiposity, hyperlipidemia and chronic gouty arthritis) and prior tendon injuries are ranked to influence the biological aspect of tendon pathogenesis by reducing the capability of the tendon to tolerate load and by modulating repair responses [84,85,86].

Finally, tendon disorders can be triggered by several extrinsic, environmental factors, which are likely to affect the stress on the tendon tissue from the outside. This includes active daily life and physically demanding workplace, poor nutrition, smoking, alcohol consumption, environmental factors (e.g., cold weather and foot wear) and pharmacological agents (e.g., fluoroquinolone and quinolone antibiotics, corticosteroids, aromatase inhibitors and statins) [87,88].

In summary, the wide-ranging variety of tendinopathy risk factors could provoke a highly diverse spectrum of local cellular response with subsequent collagen disruption and inflammation that might be one cause for the broad pathological spectrum. However, the exact mechanisms of how mechanical, intrinsic and extrinsic factors influence tendinopathy risk are largely unknown due to lack of proper models that would allow fundamental inference.

## 9. Current Tendinopathy Management Strategies

Tendon treatment often requires lengthy periods of rehabilitation, especially in the elderly population since the original biological properties and mechanical strength are rarely fully regained, and rather, frequently coupled with chronic pain. Ideally, interventions should be tailored to the actual pathology, as well as patients’ expectations. Clinical management of tendinopathy is challenging. Due to the multifactorial nature of the disease, interventions are not effective in every patient and robust and convincing evidence of treatment success for many commonly applied therapies is lacking. Classical therapeutic approaches have focused on reducing inflammation and pain, yet research suggests that little to no inflammation is present in the tendons that fail to heal.

The mainstay of first-line management of early (reactive) tendinopathies is conservative (non-operative) treatment, which comprises modification of activity, relative rest, pain relief using anti-inflammatory Non-Steroidal Anti-Inflammatory Drugs (NSAID), as well as corticosteroid injections and cryotherapy (Table 1) [78,90]. The traditional physiotherapy in combination with myofascial therapy, ultrasound, iono-and phonophoresis and acupuncture yield clinically good results, as long as no major structural damage can be observed (Table 1) [78,90]. The use of corticosteroids or NSAIDs for inflammatory suppression and pain relief is controversial based on the high risk of spontaneous tendon ruptures after local or even systemic drug delivery. Non-operative strategies also include ultrasound treatment and shock wave therapy, eccentric exercises and low-intensity laser treatment [90]. They are applied in order to promote structural remodeling and repair. However, scientific literature on non-operative treatment modalities often lacks randomization and blinding and thus, it can only be compared sparsely, due to the variety of study conditions [78,90].

Many promises are given to treatment of tendinopathies by platelet rich plasma (PRP). Not only many investigations in different tendinopathies have been undertaken, but also the technique itself is widespread meanwhile. Up to date, more than 400 growth- and immunomodulating factors are known to be enclosed in PRP. Nevertheless, current research strategies try to unveil, which factors of PRP are beneficial and which are not as not every tendinopathy and not every patient benefits from PRP injections [91,92]. Hence, PRP-based-approach for treating tendinopathy still lacks consistent results between multiple centers due to high variability of the blood derivatives and not yet precise characterization of the critical indispensable components and those that are dispensable in the PRP cocktail [92].

Many promises are given to treatment of tendinopathies by platelet rich plasma (PRP). Not only many investigations in different tendinopathies have been undertaken, but also the technique itself is widespread. Up to date, more than 400 growth- and immunomodulating factors are known to be enclosed in PRP. Nevertheless, current research strategies try to unveil, which factors of PRP are beneficial and which are not. In addition, not every tendinopathy and not every patient benefits from PRP injections [91,92]. Hence, a PRP-based-approach for treating tendinopathy still lacks consistent results between multiple centers, due to high variability of the blood derivatives and imprecise characterization of the critical indispensable components and those that are dispensable in the PRP cocktail [92].

An emerging and potent cell-based strategy for treatment of chronic degenerative tendon injuries is the autologous tenocyte implantation (Ortho-ATI) developed by Orthocell [93]. For this, the patient’s own tenocytes are extracted from healthy patellar tendon biopsy, expanded and delivered to the diseased tendon site via ultrasound guided injection under local anesthetic to stimulate tendon regeneration [94,95].

If degeneration has progressed to degenerative tendinopathy and end stage tendon rupture, scientific evidence has stated that nonsurgical treatment is less successful as only 60% of the rehabilitated tendons are functional. Dependent on anatomic location of the tendinopathy, 10%–30% of patients need to be subsequently treated by surgery after failure of conservative therapies [96]. In addition, patient immanent factors such as age, co-morbidities and local degeneration need to be taken into account, in order to offer a tailored treatment strategy.

Around 30% of Achilles tendon injuries require surgical treatment, including suturing, implantation of autologous tissue (e.g., strip of the central aponeurosis of gastrocnemius, peroneal tendon, fascia lata, flexor digitorum longus or flexor hallucis longus), an allograft or a synthetic scaffold. However, there are limitations associated with these treatment options. Autologous tissue has a limited availability and is associated with a high risk of donor site morbidity. Allografts have limited availability and are expensive due to costs associated with tissue banking, required microbiological testing and method of processing [4]. Synthetic grafts lack natural tissue organization and consequently have low bio-integration and biomechanical capacities often resulting in unsatisfactory results. After all, not only does the tendon substance need to be restored, but also the function that was lost due to the tendon rupture.

A new strategy that is being experimentally and pre-clinically explored is tendon tissue engineering, which relies on the application of effective cells injected directly to the site of tendon lesion or on the delivery of cells seeded on a suitable carrier, such as hydrogel or dense matrix in order to speed up the restorative process [97,98,99,100]. However, few treatments for tendinopathy are targeted against specific molecular processes. In most cases, there is little to no evidence of therapeutic effectiveness especially in the long term.

Cook et al. have proposed to implement the continuum model of tendinopathy to help guide targeted clinical treatments and thus to lead to improved patient outcomes [11,79]. The continuum model proposed a staging of tendinopathy based on the changes and distribution of disorganization within the tendon tissue with the aim to support clinicians to understand the various presentations of tendinopathy and to allow rational placement of clinical interventions [79]. However, this model did not integrate pain and the nervous system with the pathology. Hence, the authors proposed a revised continuum model encompassing structure, function and pain relationships, which can advise the choice of therapy; concretely, interventions addressing pain, poor function and load capacity depending on the stage of disease [79]. This study points towards the need to generate rationalized algorithms in decision making during management of tendinopathies, which may help to gain more cross-comparative study data from multiple clinical centers worldwide [79].

The current low success rate of therapeutic strategies directly reflects on one side, the deficiencies in comprehensive understanding of the molecular and cellular mechanisms controlling the tendinopathy process as well as on the other side, the further necessity of improvement in clinical management of tendinopathy by limiting the design of smart, tendon-specific strategies for countering the advancement or even preventing the onset of tendon degeneration.

## 10. Conclusions

Despite the significant progress in tendon research, to date, the management of tendinopathies is restricted to symptomatic therapy. Tendon-specific treatment options are still unavailable mainly because of our incomplete understanding of underlying molecular and cellular mechanisms and preceding risk factors. Some ongoing challenges of clinicians and researchers are identification of such prospective risk factors and early detection of the disease as well as defining effective strategies to attenuate the disease process. In the area of disease detection, innovative imaging techniques will be of great interest, whilst in the area of prevention and prophylaxis, investigating how modifying the disease risk factors might reduce the danger for developing tendinopathy and occurrence ng of tendon injury will be very relevant. The tendon research field should also pursue a more systematic way to decipher the complexity behind tendon degeneration. Research on how influencing the fate of endogenous cells and the activity and prevalence of exogenous cells affects the tendon tissue at times of homeostasis and repair, may lead to the outlining of very innovative ways to combat tendinopathies. The recent great advancement in technologies like RNA sequencing, proteomics, metabolomics, tissue engineering, nanoparticles and organ-on-chip integrated with big data analysis and computational modeling should be more strongly adopted by tendon scientists. We believe that based on joint research efforts and combining multidisciplinary knowledge and expertise, we will experience in the near future the identification of drug targetable components that counter tendinopathy or even boost tendon healing, as well as a significant innovation in cell-based therapy and in creating functional three dimensional tendon mimetics to be implemented in regenerative medicine. In turn, the long-awaited new era of improved clinical outcomes in tendinopathy management shall begin.

## Figures and Tables

**Table 1:** Current options of tendinopathy management.

Conservative Management		Surgical Management
Biomechanical Therapies	Biological Therapies	Operative Therapies
**Classical physiotherapy:** Deep transverse friction massage Myofascial manipulation Controlled motion Ultrasound (0.75-3.0 MHz; pulsed or continuous) Ionophoresis Phonophoresis Acupuncture **Electrical and laser stimulation:** Pulsed electromagnetic fields Extracorporeal shock-wave therapy Laser treatment (pulsed or continuous) **Stabilization and modification:** Taping Splinting Bracing Straps Orthotic devices **Modification of activity:** Rest Eccentric exercises **Thermic treatments:** Cryotherapy (e.g., ice packs and baths) Thermotherapy (heat)	**Pharmaceutical agents:** Anti-inflammatory drugs (NSAIDs) Systemic corticosteroids Pain control (anesthetics) Antibody therapy (e.g., IL-17, IL-1β antagonist and BMP) **Peritendinous (high volume) injections:** Corticosteroid injection Saline injection Hyaluronic acid injection Botulinum toxin (BTA) injection MMP inhibitor injection (e.g., Aprotinin) Prolotherapy Topical glyceryl trinitrate therapy Polidocanol injection Glycosaminoglycan polysulfate injection Sclerosant injection Low-dose heparin **Blood-based therapies:** Platelet-rich plasma injection Autologous blood injection Actovegin (deproteinized extract of calf’s blood) **Cell-based therapies:** Autologous tenocyte implantation (Orthocell)	Arthroscopy Debridement and decompression Endoscopic/minimally invasive surgery Percutaneous longitudinal tenotomy Radiofrequency microtenotomy Stripping and destruction of neovessels Endoscopic tendon debridement Tenolysis Gastrocnemius recession **Tendon replacement strategies after rupture:** Tendon allografts Tendon transfer Tendon prosthesis

## Abbreviations

ACL	Anterior cruciate ligament
ADAMTS	A disintegrin and metalloproteinase with thrombospondin motifs
CpG	Cytosine nucleotide followed by a guanine in the DNA along its 5′ → 3′ direction
COX2	Cyclooxygenase-2
CRABP	Cellular retinoic acid-binding protein
DNA	Deoxyribonucleic acid
ECM	Extracellular matrix
GAGs	Glycosaminoglycans
IGF	Insulin growth factor
IL	Interleukin
MMP	Matrix metalloproteinases
NF-kB	Nuclear factor ‘kappa-light-chain-enhancer’ of activated B-cells
NSAID	Non-steroidal anti-inflammatory drugs
PASTA	Partial articular supraspinatus tendon avulsion
PDGF	platelet derived growth factor
PGs	Proteoglycans
PRG4	Proteoglycan 4 or lubricin
PRP	Platelet rich plasma
RNA	Ribonucleic acid
STAT6	Signal transducer and activator of transcription 6
TGF-β	Transforming growth factor β
TIMPs	Tissue inhibitor of metalloproteinases
TNFα	Tumor necrosis factor-α
TPPP3	Tubulin Polymerization Promoting Protein Family Member 3
TSPCs	Tendon stem progenitor cells
VEGF	Vascular endothelial growth factor
